# Behaviour of the Peri-Implant Soft Tissue with Different Rehabilitation Materials on Implants

**DOI:** 10.3390/polym15153321

**Published:** 2023-08-07

**Authors:** María Baus-Domínguez, Serafín Maza-Solano, Celia Vázquez-Pachón, Marta Flores-Cerero, Daniel Torres-Lagares, María-Ángeles Serrera-Figallo, Laura Macías-García

**Affiliations:** 1 Instituto de Biomedicina de Sevilla, IBiS/Departamento de Estomatología, Facultad de Odontología, Universidad de Sevilla, C/Avicena S/N, 41009 Sevilla, Spain; mbaus@us.es (M.B.-D.); serafinmazasolano@gmail.com (S.M.-S.); celiavazpac@gmail.com (C.V.-P.); martuxifc@gmail.com (M.F.-C.); danieltl@us.es (D.T.-L.); 2 Departamento de Citología e Histología Normal y Patológica, Facultad de Medicina, Universidad de Sevilla, Avda. Sánchez-Pizjuán S/N, 41009 Sevilla, Spain

**Keywords:** histological evaluation, dental materials, gingival fibroblast, mucointegration, dental implants, polymethylmethacrylate, hybrid ceramic, reinforced composite

## Abstract

(1) Background: Mucointegration seems to gain interest when talking about success in the maintenance of dental implants. As we well know, collagen fibres cannot be inserted due to the lack of root structure on the implant surface, so the structural integration of peri-implant tissues that provide a firm seal around implants seems to be of interest when it comes to ensuring the survival of dental implants. To achieve a good epithelial barrier, the physicochemical characteristics of the surfaces of the restorative materials are of vital importance; therefore, the objective of this study is to analyse the histological behaviour of the peri-implant soft tissues in three different restorative materials. (2) Methods: Histological analysis of biopsied peri-implant keratinised mucosa, inflammatory epithelium and connective tissue in contact with a reinforced composite (BRILLIANT Crios), a cross-linked polymethylmethacrylate (TELIO CAD), and a hybrid ceramic (Vita Enamic), restored on a customised Atlantis-type abutment (Dentsply Sirona) between 60 and 180 days after restoration. (3) Results: A greater number of cells per mm2 of keratinised epithelium is observed in the reinforced composite, which could indicate greater surface roughness with greater inflammatory response. In this way, the greater number of lymphocytes and the lateral cellular composition of the inflammatory cells confirm the greater inflammatory activity towards that material. The best material to rehabilitate was hybrid ceramic, as it shows a better cellular response. (4) Conclusions: Knowing the limitations of the proposed study, despite the fact that greater inflammation is observed in the reinforced composite relative to the other materials studied, no statistically significant differences were found.

## 1. Introduction

Dental implants are currently the first-choice treatment option for the rehabilitation of patients with total or partial tooth loss. Currently, the success rate in dental implant dentistry is 96.4% (95% CI: 95.2–97.5%) at 10 years [1]. However, the long-term success of dental implants requires not only proper osseointegration but also an appropriate soft tissue arrangement in the transmucosal region, better known as mucointegration. Different studies warn of the importance of the soft tissue healing process around implants [2,3].

From a histological point of view, a proper mucosal soft tissue seal around implants consists of peri-implant epithelium and underlying connective tissue that separates the osseointegrated implant from the oral environment, thus avoiding multiple biological complications that give rise to the main implant problems of mucositis, peri-implantitis and dental implant failure [3,4,5,6,7].

Studies report that the transmucosal region of implants is much more unstable than that seen around teeth because the strength of adhesive structures, such as the internal basal lamina and the hemidesmosomes of the peri-implant mucosa is significantly weaker [3,4,7,8], as the fibres of the peri-implant connective tissue are arranged in parallel, from the vestibular/palatal/lingual periosteum in a coronal direction towards the free gingiva and without any direct connection to the restoration, as opposed to what is observed at the dental level where the fibres run perpendicular and are inserted into the cementum of the root surface [3,4,5,7,8,9,10]. Similarly, peri-implant connective tissue is up to 85% collagen and 2% cellular components compared to 60% collagen and 10% cells in periodontal connective tissue [5,7,8]. For this reason, research focused on materials and the treatment of their surfaces is currently on the rise to optimise the proliferation of fibroblasts and peri-implant soft tissue union. Among them, studies of the machined, polished, anodised titanium surface during the transgingival abutment manufacturing process or the decontamination treatments of surfaces with ultraviolet light or argon plasma [2,11,12] stand out. Especially interesting is the latter for transforming the surface into bioactive and improving cell proliferation and the quantity of adsorbed proteins [12]. However, all the studies we have found in the databases focus on finding out the cellular response, mainly in vitro and very few data in vivo, to different types of titanium surfaces for the fabrication of transepithelial prosthetic abutments or healing abutments.

There are several factors that influence the establishment and maintenance of a correct transmucosal barrier, including bacterial adhesion and the formation of pathogenic biofilms; and the materials associated with the types of abutments and dental restorations.

Several studies [4,8,9,10] show that the composition of the abutment material in contact with the peri-implant mucosa can affect it, with the abutment surface being also influential in the adhesion, proliferation and colonisation of microorganisms [2,5,13]. Titanium abutments are the “gold standard” for use in implant restorative treatments, as well as being the most studied in terms of biomechanical and biological behaviour [14,15,16]. However, the restorative abutment is not the only material that comes into contact with the peri-implant mucosa and therefore influences its composition and maintenance. For this reason, the aim of this study is to evaluate how the type of dental restorative material chosen influences the formation of adhesive structures critical for the proper sealing of the peri-implant mucosa since studies have shown that the topography of the surface of the transmucosal component influences the formation of the biologic width [6,7,13], up to now it has not been known how and to what extent the restorative material can influence its maintenance.

Given that there are currently many studies analysing the behaviour of peri-implant tissues around definitive restorative materials such as ceramics or zirconium oxide, in this research, we focus on comparing restorative materials in temporary restorations made of a BRILLIANT Crios reinforced submicron hybrid composite (COLTENE, Altstätten/Switzerland); a hybrid ceramic with a dual ceramic-polymer matrix (Vita Enamic^®^ (VITA, Zahnfabrik H. Rauter GmbH & Co. KG, Spitalgasse 3, D-79713 Bad Säckingen, Germany); and cross-linked polymethylmethacrylate (PMMA) (Telio CAD^®^ (Ivoclar Vivadent AG, Schaan, Liechtenstein)) on customised Atlantis-type titanium nitride abutments from Dentsply Sirona.

The null hypothesis of this study is that peri-implant tissue behaves worse on materials with poorer surface characteristics due to greater bacterial accumulation.

## 2. Materials and Methods

### 2.1. Type of Study

This is a prospective observational cohort study approved by the Andalusian Biomedical Research Ethics Coordinating Committee (Code US-DTL-2022.1) that complies with all the guidelines of the World Medical Association Declaration of Helsinki: ethical principles for medical research involving human subjects [17].

This is an observational study where the only invasive procedure was the biopsy of the peri-implant tissues of those implants rehabilitated with the materials to be studied.

All patients received information about the study and gave informed consent to both the procedure in question and their participation in the study.

### 2.2. Samples

The aim of the study is to perform a histological analysis of the keratinised epithelium, inflammatory activity and connective tissue of three different rehabilitation materials on individual Atlantis-type abutments made of titanium nitride from the company Dentsply Sirona.

For this, the inclusion criteria that were followed were:

- Patients older than 18 years of age.
- Patients who need rehabilitation with implants in the posterior sectors, from first premolars to second molars, both in the upper and lower arches.
- Patients with no medical contraindication to implant surgery.
- Patients without active periodontal disease.

The exclusion criteria were smoking patients (≥10 cigarettes/day), patients undergoing treatment that altered bone metabolism or soft tissue healing, patients with poor oral hygiene, patients with uncontrolled periodontal disease, and patients with alcoholism or drug addiction problems.

### 2.3. Elaboration and Fitting of the Prosthetic Restoration

The implants, placed by a specialist, were Astra EV (Astra Tech Implant System EV. Dentsply Sirona, S.A., Barcelona, Spain), with up to 3 different diameters: 3.6, 4.2 and 4.8. They were bone-level implants with internal conical connections.

All implants were placed by the same professional. After implant placement, a healing abutment was screwed according to the gingival height of the patient. The implants were rehabilitated 3 months after placement.

**Figure 1:**
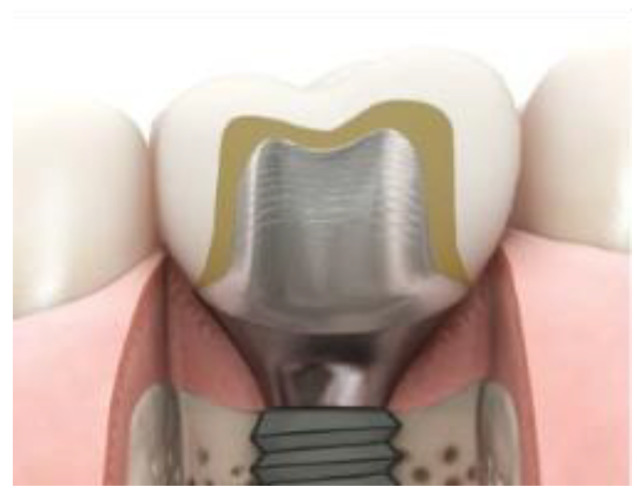
ATLANTIS patient-specific abutment. Beyond CAD/CAM ATLANTIS^TM^ patient-specific abutments available on https://www.dentsplysirona.com/es-ib/productos/implantes/soluciones-digitales/pilares-atlantis.html, accessed on 15 January 2023.

The impressions for preparing the restoration were taken digitally using the Primescan™ intraoral scanner (Software Connect 5.1, Dentsply Sirona, S.A. Barcelona, Spain). The rehabilitation was designed on the Atlantis custom abutment with a minimum subgingival depth of 2 mm to allow the restorative material to have contact with the peri-implant tissue (Figure 1).

All abutments were screwed at the torque recommended by Dentsply Sirona (35 Ncm^2^) directly to the implant. The crowns were cemented on the interfaces with a temporary self-curing zinc oxide-eugenol cement so that the restoration could be removed later without damaging or altering the cellular composition of the soft tissue attachment.

After the placement of the prosthetic crown, all patients received individual verbal and written oral hygiene instructions. The clinical brushing technique was reinforced, and the use of a medium bristle brush was recommended. All patients were monitored at their monthly visits until a biopsy was taken to ensure that their periodontal health was maintained throughout the follow-up study. Similarly, at each visit, it was evaluated that the crowns were correctly screwed (that there was no mobility).

### 2.4. Description of Restorative Materials

BRILLIANT Crios (COLTENE, Altstätten, Switzerland): this is a reinforced submicron hybrid composite comprising a barium glass of less than 1.0 μm in size, amorphous silica oxide of less than 20 nm in size, a cross-linked methacrylate resin matrix and inorganic pigments (ferrous oxide or titanium dioxide). It has the characteristics of a composite such as high flexural strength and a modulus of elasticity similar to that of a tooth. Its advantages include the fact that it does not require firing and can therefore be modified and repaired in situ.

Vita Enamic^®^ (VITA, Zahnfabrik H. Rauter GmbH & Co. KG, Spitalgasse 3, D-79713 Bad Säckingen, Germany): this is a pre-sintered fine-structured feldspathic ceramic with a porous form (86% by weight) infiltrated with a polymer (14% by weight) that has the advantage of having an elastic modulus about 50% lower compared to other feldspathic ceramics, which makes it closer to that of dentine. They are also easier to mill and fit, as well as repair from composite resins. The material is considered a resin-ceramic hybrid, as it is composed of two interconnected networks (dominant ceramic + polymer). Although it is marketed as a polymer-infiltrated ceramic, scientific analysis shows that the inorganic matrix is rather an amorphous glass.

Telio CAD^®^ (Ivoclar Vivadent AG, Schaan, Liechtenstein): cross-linked polymethylmethacrylate (PMMA) whose acrylic sheet is obtained from the polymerisation of methyl methacrylate. This study is based on a millable polymethylmethacrylate disc with double cross-linking. Its properties include transparency of around 93%, being the most transparent of all plastics, with high impact resistance, resistance to weathering and ultraviolet rays. Likewise, it is an excellent thermal and acoustic insulator, it is very light, being only slightly heavier than water, it has a hardness similar to that of aluminium, it is very easily repaired with a polishing paste and can be cold machined, but not bent.

The BRILLIANT Crios and Vita Enamic^®^ restorations were milled on the inLab MC XL milling machine from Dentsply Sirona, whereas the PMMA crowns were milled on the inLab MC X5 milling machine from Dentsply Sirona.

All rehabilitations were surface treated in the same way. First, a polishing was carried out with a silicone disc (Edenta Exa Cerapol UM, EDENTA AG, Lusteaneu, Switzerland) together with a mounted goat hair disc brush and finally with a felt disc.

### 2.5. Biopsy

All biopsies were taken from the rehabilitated areas at least 60 days after the restoration had been placed, specifically, around 75 days on average.

Biopsies were taken under local anaesthesia (lidocaine 2% and epinephrine 1:100,000, Xilonibsa, Inibsa, Barcelona, Spain). The peri-implant tissue samples were 1 to 2 × 4 to 5 mm obtained by external bevel incision with a 15C scalpel blade, encompassing the vestibular epithelium in contact with the restorative material.

The samples were coded during pathological processing to allow a single-blind histological analysis. Both the crowns and biopsied tissue were placed in a sterilised 40% formalin analysis jar for histological analysis.

Biopsies were taken from 21 January 2021 to 28 July 2021.

Finally, after the biopsies were taken, the patient was fitted with a final zirconium oxide restoration (IPS e.max ZirCAD™ Multi, Ivoclar Vivadent AG, Schaan, Liechtenstein) on the customised ATLANTIS abutment that was already screwed onto the implant.

All patients came back one month after the biopsy to check that the area was healing.

### 2.6. Histological Analysis

The gingival specimens consisted of 4-mm formalin-fixed, paraffin-embedded sections.

**Figure 2:**
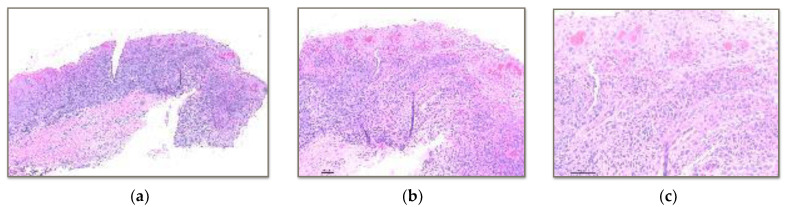
(**a**) 4× histology image with haematoxylin-eosin technique of the severe lymphocytic inflammatory infiltrate located in the intermediate zone of the soft tissue in contact with the reinforced composite (BRILLIANT Crios). (**b**) 10× image showing effacement of the basement membrane and the mucosal-submucosal junction. As well as vascular congestion and the presence of exocytosis. (**c**) 20× image showing papillary congestion and the presence of exocytosis.

**Figure 3:**
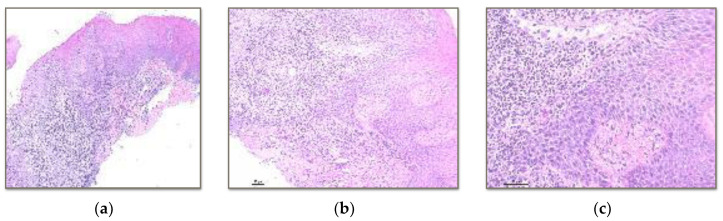
(**a**) 10× histology image with haematoxylin-eosin technique of the severe lymphocytic inflammatory infiltrate located in the intermediate zone and extending deep into the soft tissue in contact with the reinforced composite (BRILLIANT Crios). (**b**) 10× image showing effacement of the basement membrane and the mucosal-submucosal junction. (**c**) 20× image showing very mild exocytosis.

**Figure 4:**
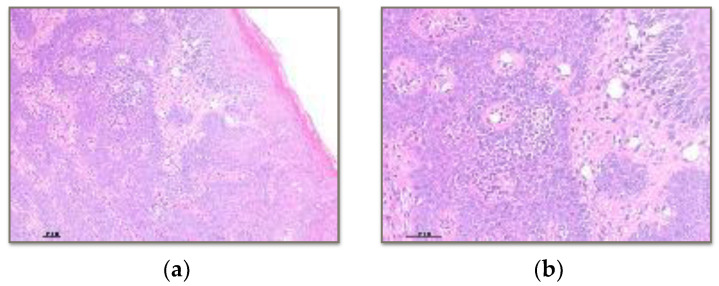
Histology with haematoxylin-eosin technique of soft tissue in contact with polymethylmethacrylate (Telio CAD^®^). (**a**) 10× image of stratified squamous epithelium with intercellular lymphocytic infiltration. (**b**) 20× image showing the presence of intraepithelial lymphocytes between the cell spines.

The samples were evaluated with haematoxylin and eosin and Masson’s trichrome histology. Haematoxylin and eosin staining allowed for the recognition of epithelial changes and vascular and cellular components (Figure 2, Figure 3 and Figure 4).

On the other hand, Masson’s Trichrome staining was used for detecting and distributing collagen fibres.

For documentation purposes, all processed images were digitally photographed (Colour Camera Nikon DS-Fi3, v. 100.06.3307.E9 (Nikon Inc., Tokyo, Japan) using an OLYMPUS VANOX AHBT3 (Olympus Corp., Tokyo, Japan) optical microscope with 4×, 10×, 20× and 40× objective lenses.

The NIS Elements Imaging Software (v. 5.21.00) was used for image digitisation and processing ((Nikon Inc., Tokyo, Japan).

### 2.7. Statistical Analysis

#### 2.7.1. Descriptive Analysis

A full descriptive analysis was conducted detailing all variables.

The sample includes patients for whom more than 60 and less than 180 days have elapsed between provisional crown placement and the biopsy.

#### 2.7.2. Normality of Numerical Variables

The Kolmogorov-Smirnov test was applied to determine normality.

#### 2.7.3. Cross-Tabulation of Material Type and Qualitative Variables

The Chi^2^ test was performed. To determine the groups that make the difference, the Habermann corrected typed residuals were used to obtain the significance of the cells independently.

#### 2.7.4. Cross-Tabulation of Material Type and Numerical Variables

ANOVA was applied for the normally distributed variables and Kruskal-Wallis for the rest.

## 3. Results

### 3.1. Patients and Characteristics of Their Restorations

- Vita Enamic^®^: 11 patients (11 crowns)
- BRILLIANT Crios: 10 patients (10 crowns)
- Telio CAD^®^: 13 patients (13 crowns)
- A total of 34 restorations were evaluated.

### 3.2. Analysis of the Materials with Respect to Qualitative Variables

#### 3.2.1. Predominant Location of Inflammatory Activity in the Epithelium

- Vita Enamic^®^: 100% in the intermediate zone.
- BRILLIANT Crios: 100% in the intermediate zone (Figure 2 and Figure 3).
- Telio CAD^®^: 100% in the intermediate zone

#### 3.2.2. Cellular Composition of Inflammatory Activity

- Vita Enamic^®^: 70% lymphocytes and 30% polymorphosnuclear.
- BRILLIANT Crios: 100% lymphocytes.
- Telio CAD^®^: 85% lymphocytes and 15% polymorphosnuclear (Figure 4).

#### 3.2.3. Lateral Cellular Composition of Inflammatory Activity (Table 1)

- Vita Enamic^®^: 18% no cells, 18% lymphocytes, 9% lymphocytes and plasma cells; 18% polymorphonuclear, 10% polymorphonuclear and lymphocytes; and 27% polymorphonuclear and plasma cells.
- BRILLIANT Crios: 60% polymorphonuclears and 40% polymorphonuclears and plasma cells.
- Telio CAD^®^: 8% are no cells, 8% are lymphocytes, 8% are lymphocytes and plasma cells, 54% are polymorphonuclear, and 22% are polymorphonuclear and plasma cells.

#### 3.2.4. Number of Collagen Fibres in Connective Tissue

The data are summarised in Table 2.

#### 3.2.5. Arrangement of Connective Tissue Collagen Fibres

**Figure 5:**
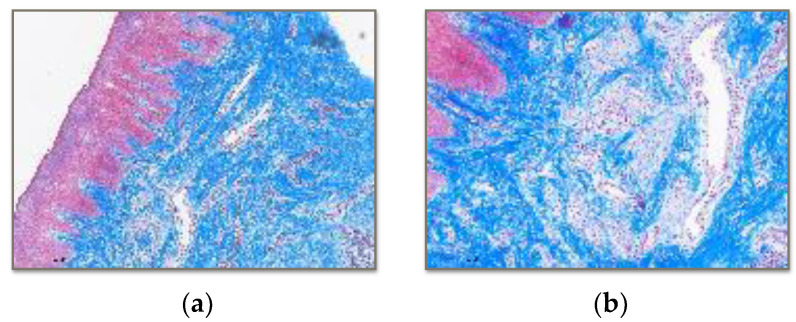
Histology with Masson’s Trichrome technique of soft tissue in contact with BRILLIANT Crios. (**a**) 4× image showing proliferation of connective tissue bundles without alterations in their arrangement in the submucosal space. (**b**) 10× image showing increased vascular proliferation.

One hundred percent of the collagen fibres are arranged normally in the cases of Vita Enamic and BRILLIANT Crios (Figure 5). However, in the case of PMMA, 90% of the fibres are arranged normally, but 10% of the fibres are observed in altered positions.

#### 3.2.6. Vascular Proliferation of Connective Tissue

The data are summarised in Table 3.

### 3.3. Analysis of the Materials with Respect to Quantitative Variables

The data are summarised in Table 4.

## 4. Discussion

Currently, a high survival rate is observed for implant-supported restorations (97.2% for 5 years and 95.2% for 10 years). However, the prevalence of peri-implantitis two years after completion of placement is 34% of patients and 21% of implants [4]. Therefore, nowadays, to speak of success in dental implantology, it is not enough to achieve correct osseointegration but rather a healthy integration of the peri-implant soft tissue and the absence of an increased inflammatory response [18].

The peri-implant mucosa has a substantially different histology and arrangement from the healthy soft tissue around the teeth since the epithelial seal is established with a significantly lower number of hemidesmosomes and the internal basal lamina, which reduces on a large scale the force that binds the epithelium [3]; this, together with the parallel arrangement that the collagen fibres take, make the peri-implant mucosa simply conform around the implant abutment and crown, without any biological attachment.

This prospective cohort study has been specifically designed to analyse on a human model three different restorative materials (BRILLIANT Crios (COLTENE, Altstätten, Switzerland), Vita Enamic^®^ (VITA, Zahnfabrik H. Rauter GmbH & Co. KG, Spitalgasse 3, D-79713 Bad Säckingen, Germany) and Telio CAD^®^(Ivoclar Vivadent AG, Schaan, Liechtenstein) cemented on customised titanium nitride Atlantis-type abutments by Dentsply Sirona.

It should be noted that there is very little updated literature available that reports on the response and/or composition of the peri-implant transmucosal region of implants rehabilitated with these materials. In fact, no in vivo or in vitro study has been found that evaluates the state and composition of the soft tissues around implants with restorations made with these materials. The vast majority of published studies report results on the cellular response in titanium with different surface treatments. For example, the multicenter randomised controlled trial by Pera et al. 2022 [2] investigated whether CrN/NbN (superlattice) coating on a titanium abutment significantly influenced the behaviour of the peri-implant tissue when compared to traditional machined abutments; however, no statistically significant differences were observed. The systematic review of in vitro studies by Corvino et al. 2020 [11] stated that zirconia, collagen plating, electropolishing, plasma cleaning, and laser dimpling promote better cell behaviour compared to machined titanium. Results can be complemented with the immunohistochemical study by Mangano et al. (2018) [6], who report a significantly lower inflammatory infiltrate in direct metal sintering laser (DMSL) additive manufacturing healing caps compared to machined ones. Like these, there are many more articles reporting promising results in terms of a better biological response of cells and, therefore, tissues, such as the systematic review of in vitro studies by Carossa et al. (2022) [12] who states that plasma argon treatment seems to be a good resource for improving cell adhesion and protein adsorption.

The new techniques aimed at improving the formation of the peri-implant transmucosal barrier are still interesting, but it should not be forgotten that the peri-implant tissues are not only in contact with a transepithelial prosthetic abutment but also with the restorative materials used for the making crowns, in this case provisional, especially used for rehabilitation cases in the aesthetic sector.

The method described in this manuscript aims to determine which of the three materials influences a worse establishment of the transmucosal barrier based on an immunohistochemical analysis of the peri-implant soft tissue in contact with the supragingival components made of the three different materials described above. For this purpose, different variables related to inflammation will be studied, such as the predominant location and composition of inflammatory activity, the number and arrangement of collagen fibres and connective tissue vascularisation.

The surgical protocol, as well as the reviews and follow-ups of the patients until the placement of their definitive crowns and subsequent reviews, passed without incident. The use of these customised abutments eliminates the variability of the abutment’s influence when comparing different materials used to manufacture the crowns. Similarly, despite the different materials that could be used to manufacture prosthetic abutments, titanium nitride was chosen because a recent systematic review and meta-analysis [16] found it to be the only material where no changes in marginal bone loss (MBL) were observed over time.

The connective tissue around the implants contains fibroblasts, endothelial and immune cells. Fibroblasts are one of the most studied cell types, as they are the main cells responsible for the secretion, remodelling and orientation of collagen fibres, which, as explained above, are arranged parallel to the implant surface, constituting a weaker biological seal than that found around the teeth [3,4,5,7,8,9,10]. This seal is maintained by mere physical adaptation rather than the biological fixation observed around the teeth [4,11,19].

Many researchers note that the behaviour of these fibroblasts may be affected by the surfaces not only of the implant but also of the prosthetic attachments, including the transmucosal attachment and the restoration, whereby the microtopography, roughness, wettability, etc., of the material, may influence the arrangement of these collagen fibres and thus the soft tissue seal around the implants [4,8,16,18,20,21,22]. In turn, the materials seem to show an extensive influence on the behaviour of the epithelial cells.

A proper transmucosal seal seems to be guided by the topography of the material in contact with the peri-implant soft tissues [4,8,16,18,19,20,21,22,23,24]. Among the main characteristics that seem to be important in the process, roughness and hydrophilicity are highlighted.

With regard to roughness, it was previously accepted that the higher the surface roughness, the more favourable it would be for fibroblast and epithelial cell extension and proliferation [8,18]. However, the impact of surface roughness is currently controversial [8,20]. Studies have reported that a rough surface can positively influence cell attachment [6,7] by improving the quantity and quality of hemidesmosome formation [6]. However, the literature does not clarify their preference on whether a rough or smooth transmucosal portion is better [6]. On the one hand, it is not clear that roughness can influence fibre arrangement [8]; other studies report that soft tissue adhesion is not significantly influenced by the roughness of the material, while other histological studies did show the influence and claimed that moderate roughness could be beneficial [2,6,20]. Other authors claim that a smooth topography is more favourable for epithelial cells [7,18,23]. Moreover, surface roughness may have a greater influence on bacterial adhesion [16,19]; however, no effect of surface topography on either BoP or bone levels was observed [20].

Some studies indicate that roughness favours soft tissue immunoreactivity in the area around the implant [8]. Under this controversy, a threshold of 0.4μm is established that should not be exceeded, as it implies a noticeable increase in roughness that increases the affinity of microorganisms [19,24]; however, others set a roughness of <0.8 μm to ensure less bacterial colonisation [21,23] and <0.2 μm to define smooth walls [21]. Some studies suggest that smooth surfaces could reduce the risk of bacterial colonisation; however, despite working with implants with machined surfaces and smooth transmucosal components, the incidence of peri-implantitis has not been significantly reduced [6]. The problem probably resides in the fact that most of the studies deal with in vitro experiments, which are difficult to transfer to the complexity of the mechanics and microbiome of the oral cavity.

In our immunohistochemical analysis, it is confirmed that cell adhesion, proliferation and collagen fibre release varied depending on surface roughness and point in time.

The highest number of cells per mm^2^ observed in the keratinised epithelium was 41.30/mm^2^ for the reinforced composite (BRILLIANT Crios, Coltene Holding, Altstätten, Switzerland), 37.00/mm^2^ for the PMMA (Telio CAD^®^, Ivoclar Vivadent AG, Schaan, Liechtenstein) and 28.73/mm^2^ for the polymer-infiltrated ceramic (Vita Enamic^®^, VITA, Zahnfabrik H. Rauter GmbH & Co. KG, Spitalgasse 3, D-79713 Bad Säckingen, Germany). Moreover, 100% were lymphocytes for BRILLIANT Crios, compared to 70% lymphocytes in Vita Enamic^®^ and 85% in Telio CAD^®^. Thus, a greater inflammatory response is observed for the reinforced composite but without statistically significant results.

It is clear that the material that seems to favour a better behaviour of the peri-implant tissue is the ceramic infiltrated with Vita Enamic^®^ polymer, as it is the material in which a histological analysis shows a lower inflammatory cellular content, a mostly normal amount of collagen fibres (80% of the cases) in the proper arrangement and a normal vascular proliferation in 73% of cases. However, the results are not very different from those reported in the case of Telio CAD^®^ (PMMA), which show a similar inflammatory cellular composition to Vita Enamic^®^, a connective tissue with a mostly normal number of collagen fibres (69% of the cases) with 90% adequate arrangement (as opposed to 10% altered) and 85% normal vascular proliferation.

These results seem to be in line with other research where PMMA is studied together with resin composites, such as a study by Chokaree P. et al. [21], where it is observed that resin composites, despite showing increased gingival epithelial attachment, are associated with a pronounced accumulation of plaque that can lead to mucosal inflammation, as reported in our histology.

Similarly, this review [21] shows how CAD/CAM-made PMMA, such as the PMMA studied in our case, is a suitable material for temporary restorations as it facilitates the correct maturation of peri-implant tissues due to its low surface roughness, similar to that observed in some ceramics [21].

Hydrophilicity or wettability is a characteristic that influences the adhesion of proteins and, therefore, an important mechanism for the proper establishment of a seal. Research has shown that the effect of wettability may increase the proliferation and migration of fibroblasts and epithelial cells [2,6,8,12,18,20] as less cell spreading towards hydrophobic materials is observed [18]. Surface treatments such as Argon Plasma increase wettability and improve cell adhesion, not only of fibroblasts but also of osteoblasts, by activating the treated surface, thus facilitating cell propagation and protein adsorption [12]. However, it should not be forgotten that hydrophilic materials have a positive correlation with bacterial plaque accumulation [21].

Likewise, CAD/CAM PMMA is considered hydrophobic but shows more fibroblast binding than self-curing material [21,22,23], as well as less residual monomer release and, therefore, higher biocompatibility [21,22].

This leads us to believe that CAD/CAM PMMA is a good material for the manufacture of implant-supported provisional restorations, not only because it seems to favour proper healing and maintenance of the peri-implant mucosa but also because of its ease of manufacture, fit and low cost compared to other materials, as no statistically significant differences were observed in this study that would make it necessary to opt for a single material.

Therefore, it could be said that the null hypothesis proposed in this study is accepted since a worse behaviour of the peri-implant mucosa is observed in the material with worse surface characteristics, although the differences are not statistically significant.

Among the limitations of the study is the relatively small size of the sample studied, as well as the fact that all biopsied samples correspond to the same moment of healing/maintenance of the peri-implant mucosa and different time points (mean 75 days) were not considered.

The final objective of this study is to provide information about the effect that different materials can have on the formation and integration of the peri-implant transmucosal barrier in order to facilitate the choice of optimal materials for the manufacture of both temporary and permanent restorations since the transmucosal pillars are not the only element in contact with the soft tissues around the implants, these prosthetic pillars being the main study objective of most of the published literature.

Choosing the right material for dental implant restorations seems to influence not only the initial healing of the peri-implant tissue but also its maintenance and, thus, the long-term survival of the implant.

## 5. Conclusions

Although materials for the restoration of implant-supported prostheses are continuously being studied with the aim of understanding the behaviour of the peri-implant soft tissue to ensure and maintain a correct mucosal seal around the implant, there is currently no recent scientific evidence available that analyses the three materials studied in this study in vivo in humans.

According to the results obtained and taking into account the limitations of the present study, no statistically significant differences were found in terms of composition and behaviour of the peri-implant soft tissue in contact with the crowns made of the different materials studied (BRILLIANT Crios, Vita Enamic^®^ and TelioCAD^®^). However, BRILLIANT Crios reinforced submicron hybrid composite restorations reveal worse cell responses than Vita Enamic^®^ hybrid ceramic and Telio CAD^®^ cross-linked polymethylmethacrylate in terms of greater inflammatory response.

Further studies are needed to help reach more specific conclusions about which material has a more positive influence on the establishment and maintenance of the transmucosal barrier around dental implants.

## Figures and Tables

**Table 1:** Lateral cellular composition of inflammatory activity.

Cell Composition	Vita Enamic^®^	BRILLIANT Crios	Telio CAD^®^
No cells	18	0	8
Lymphocytes	18	0	8
Lymphocytes and Plasma Cells	9	0	8
Plasma Cells	0	0	0
Plasma Cells, Polymorphonuclears and Eosinophils	0	0	0
Polymorphonuclears	18	60	54
Polymorphonuclears and Eosinophils	0	0	0
Polymorphonuclears and Lymphocytes	10	0	0
Polymorphonuclears and Plasma Cells	27	40	22

All numbers refer to the percentage of cells observed in the total number of histological sections of the biopsies of each material studied.

**Table 2:** Number of collagen fibres in connective.

Collagen Fibres	Vita Enamic	BRILLIANT Crios	Telio CAD^®^
Diminished	0	20	8
Normal	80	30	69
Increased	20	50	23

All numbers refer to the percentage of fibres observed in the totality of histological sections of the biopsies of each material studied. It is considered diminished when less than 60% of collagen fibres are observed, “normal” when between 60–80% of collagen fibres are observed and increased if more than 80% of collagen fibres are observed.

**Table 3:** Connective tissue vascular proliferation.

Collagen Fibres	Vita Enamic	BRILLIANT Crios	PMMA
Diminished	9	20	8
Normal	73	50	85
Increased	18	30	8

All numbers refer to the percentage of blood vessels in the total histological sections of the biopsies of each material studied. It is considered “diminished” when less than 35% vascular proliferation is observed, “normal” when around 35% is observed and increased if more than 35% vascular proliferation is observed.

**Table 4:** Summary of quantitative variables according to material.

Variable	Vita Enamic^®^	BRILLIANT Crios	Telio CAD^®^
Keratinised Epithelium. Thickening (mm)	0.80	0.76	0.59
Keratinised Epithelium. Epithelium ridge (mm)	7.82	8.60	7.15
Keratinised Epithelium. Exocytosis. Number of lymphocytes per mm^2^	18.27	41.30	37.00
Keratinised Epithelium. Exocytosis. Number of polymorphonuclears per mm^2^	10.45	0	0
Keratinised Epithelium. Exocytosis. Total number of cells per mm^2^	28.73	41.30	37.00
Keratinised Epithelium. Parakeratosis. Number of cores per mm^2^	36.82	40.80	50.38
Non-Keratinised Epithelium. Thickening (mm)	0.53	0.49	0.32
Non-Keratinised Epithelium. Epithelium ridge (mm)	3.67	3.29	4.40
Inflammatory activity. Intensity. % Inflammatory cells	39.09	39.50	30.58
Connective tissue. Quantitative analysis. Number of papillas	6.45	7.80	6.31
Days between placement and sample collection	74.73	75.60	70.08

## Data Availability

Data available on request due to restrictions.
